# Unique Gender-Related Characteristics and Differences of Chronic Myeloid Leukemia Patients in Azerbaijan: A Retrospective Cohort Study

**DOI:** 10.7759/cureus.94677

**Published:** 2025-10-15

**Authors:** Aytan Shirinova, Chingiz Asadov, Aypara Hasanova, Zohra Alimirzoyeva

**Affiliations:** 1 Hematology, National Hematology and Blood Transfusion Center, Baku, AZE; 2 Genetics, National Hematology and Blood Transfusion Center, Baku, AZE

**Keywords:** abl kinase mutations, chronic myeloid leukemia, cml epidemiology, imatinib response, molecular monitoring, sex differences, survival, tyrosine kinase inhibitors

## Abstract

Objective: The objective of this study is to evaluate gender-related demographic, clinical, and molecular differences, and imatinib response in patients with chronic myeloid leukemia (CML) in Azerbaijan.

Setting: The study was carried out at National Hematology and Blood Transfusion Center, Baku, Azerbaijan.

Period: This is a retrospective data analysis carried out from January 2008 to December 2023.

Methods: A total of 763 patients with Ph+ CML were included. Case records were reviewed for demographic data, disease phase, risk group, clinical manifestation, molecular characteristics, and response to imatinib. Genetic mutations were analyzed in 163 patients.

Results: A total of 763 CML patients were analyzed, with a female predominance (54%) observed, an atypical trend compared to global data. Female patients had significantly smaller spleen sizes, lower hemoglobin, and higher platelet counts than male patients. Imatinib response was better in female patients (55.2%) compared to male patients (44.8%) (p = 0.03). ABL kinase domain mutations were significantly more frequent in male patients (21.3%) than in female patients (six percent) (p = 0.004), with T315I mutation also higher in male patients (p = 0.036). Survival analysis showed significantly better overall survival in female patients (p = 0.038), although subgroup differences by risk category were not statistically significant.

Conclusion: This study highlights significant gender-related differences in CML patients in Azerbaijan, including a unique female predominance, better treatment response, and superior survival outcomes in women. These findings underscore the need to consider gender as a relevant factor in CML prognosis, treatment planning, and future research.

## Introduction

Sex-related differences in disease presentation and outcomes are increasingly recognized across many medical fields, including hematological malignancies like chronic myeloid leukemia (CML) [1-3]. Historically, male predominance has been reported in CML, and younger female patients have shown favorable prognostic features according to the Sokal score [4]. Despite these observations, systematic evaluations of sex-related differences in CML remain limited.

CML is a clonal myeloproliferative neoplasm characterized by the Philadelphia chromosome, arising from the BCR-ABL1 fusion gene formed by translocation t(9;22). Its incidence is approximately one to two cases per 100,000 adults annually, accounting for around fifteen percent of adult leukemia cases [5]. The disease progresses from chronic to accelerated and blastic phases. The introduction of tyrosine kinase inhibitors (TKIs) such as imatinib has transformed CML into a manageable chronic illness, achieving 10-year survival rates of nearly 80 percent [6].

Earlier studies suggested that women with CML presented with more adverse risk factors but experienced better survival during the interferon treatment era [7]. However, whether sex continues to affect clinical and molecular features in the modern TKI era remains unclear. During the TKI era, although the incidence of TKI switching is higher among women based on data from the SIMPLICITY study [8], there is no strong evidence of sex disparities in the efficacy of TKIs with regard to achievement of deep cytogenetic and molecular remissions. Similarly, no sex-related differences in the overall survival of CML patients during the TKI era have been demonstrated [9]. Based on the sex-related differences in the presentation and outcomes of CML patients before the use of TKIs, it could be hypothesized that male sex may be implicated in the acquisition of secondary molecular events driving disease progression.

Imatinib resistance occurs in a substantial subset of patients through BCR-ABL domain mutations, gene amplifications, or changes in drug metabolism pathways [10]. The cytochrome P450 (CYP) system, particularly isoforms like CYP3A4, CYP3A5, and CYP2C8, plays a key role in imatinib metabolism, and variations in CYP genes may influence treatment outcomes [11].

The rationale for this study is threefold: first, to clarify the role of sex in CML outcomes in the TKI era, where sex-based survival differences remain underexplored; second, to assess the potential biological contribution of sex-related variability in TKI metabolism through the CYP system; and third, to provide population-specific epidemiological and clinical data from Azerbaijan, where unique demographic and environmental factors may influence disease characteristics and outcomes. These considerations underscore the need for our nationwide retrospective analysis.

Given the growing interest in gender differences in disease onset and prognosis, we analyzed sex-related patterns among CML patients in Azerbaijan.

Study objectives

The objectives of this study were to evaluate sex-related demographic and clinical characteristics of CML patients in Azerbaijan, compare imatinib response and survival outcomes between male patients and female patients, assess molecular differences including ABL kinase domain mutations and CYP polymorphisms, and provide a regionally specific analysis to determine whether sex should be considered a relevant factor in CML prognosis and management.

## Materials and methods

This retrospective study included CML patients treated at the National Hematology and Blood Transfusion Center of Azerbaijan between January 2008 and December 2023. We reviewed the medical records of 763 patients with confirmed Ph+ CML. Patients were included only if complete demographic data (age, sex, residence), baseline clinical characteristics (spleen size, hemoglobin, white blood cell count, platelet count), and treatment initiation details were available. Patients with missing or incomplete information in any of these categories, or with non-Ph+ CML, were excluded. Sex was defined according to the designation recorded in patients’ medical records, which is based on official identity documents at the time of diagnosis. All analyses were performed using this binary classification (male vs. female). Disease phase was classified per European LeukemiaNet criteria, as well as diagnosis and management [12].

Molecular analyses included BCR-ABL1 (p210) fusion transcript detection using reverse transcription-quantitative PCR, with e13a2 and e14a2 isoforms analyzed from peripheral blood or bone marrow. For a subset of 163 patients, ABL kinase domain mutations and CYP polymorphisms were evaluated using standard molecular protocols.

Statistical analyses were performed with IBM SPSS Statistics for Windows, Version 31 (Released 2025; IBM Corp., Armonk, New York, United States). Chi-square and Fisher’s exact tests assessed categorical data, while logistic regression was used to estimate odds ratios with 95% confidence intervals. The significance threshold was set at p < 0.05. Ethical approval was granted by the National Hematology and Blood Transfusion Center’s research committee, and all patients provided written informed consent.

For survival analysis, the index date was defined as the date of confirmed CML diagnosis at the National Hematology and Blood Transfusion Center. Overall survival (OS) was calculated from this index date until death from any cause or last follow-up. Patients alive at last contact were censored at that time point. The Kaplan-Meier method was used to estimate OS, and comparisons between sexes were performed with the log-rank test. No progression-free survival endpoint was analyzed in this study.

## Results

Incidence and prevalence

**Figure 1:**
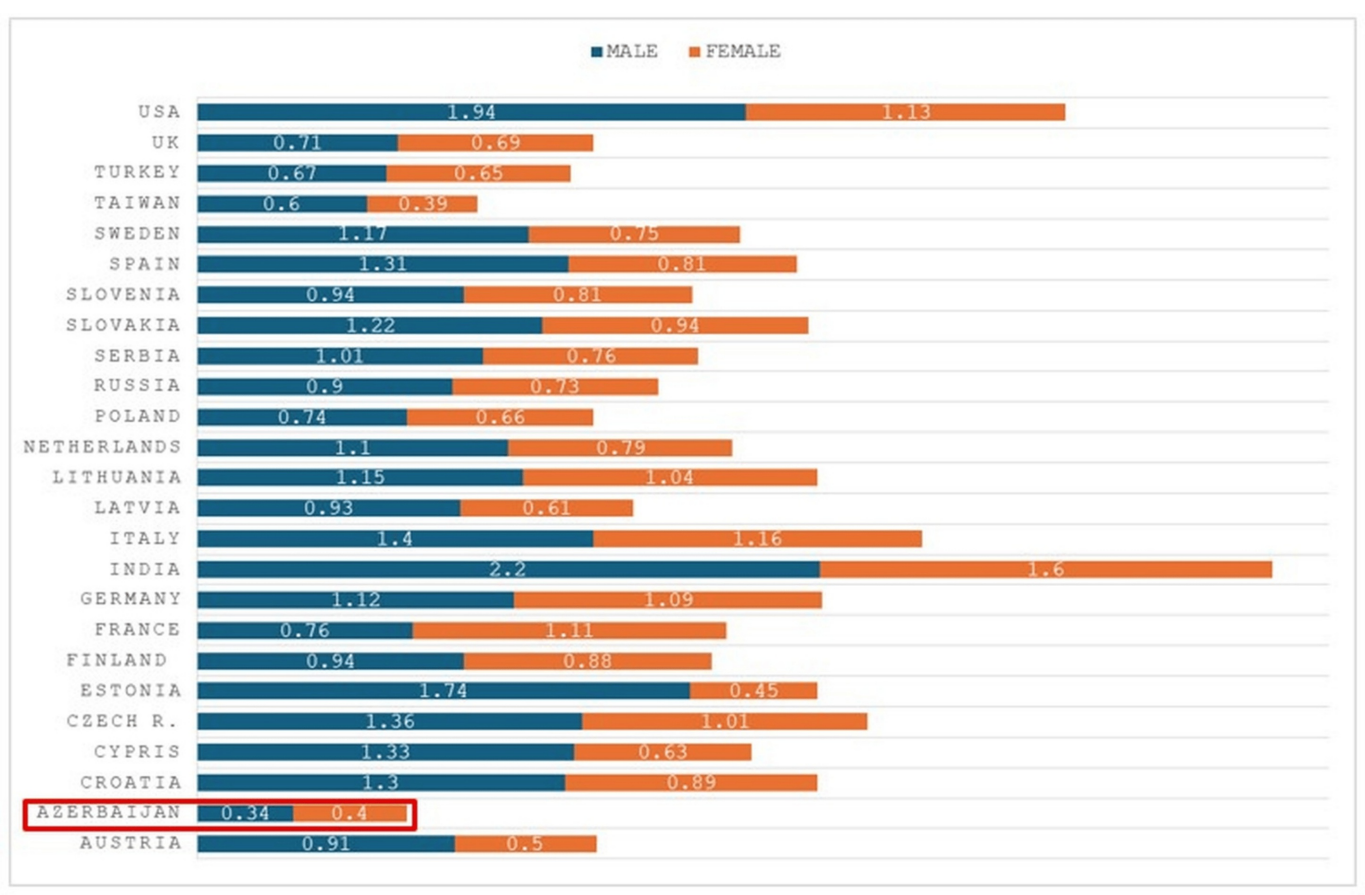
Incidence of chronic myeloid leukemia (CML) in male patients and female patients across different countries. The horizontal bar chart compares sex-specific incidence rates of CML (per 100,000 population) in 24 countries, including Azerbaijan (highlighted in red). Bars represent incidence rates; corresponding values are given as rates, not percentages of total cases. Blue bars represent male incidence and orange bars represent female incidence. Data are presented as incidence rates calculated from available national and regional registries, expressed as N and % of the population at risk. In most countries, male predominance is observed, while Azerbaijan demonstrates a higher incidence in female patients. In Azerbaijan, incidence was 0.41 per 100,000 in female patients and 0.34 per 100,000 in male patients (as shown in the figure).

**Figure 2:**
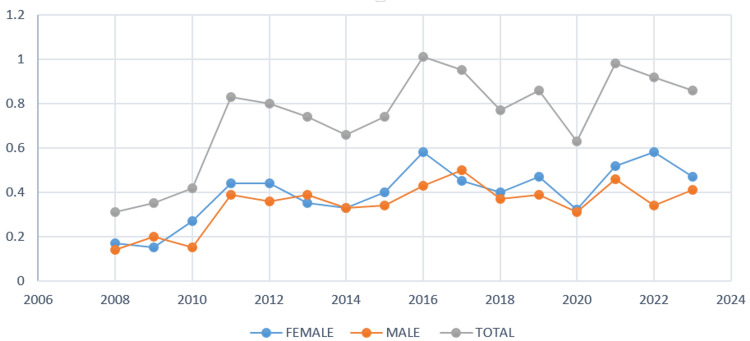
Annual incidence of chronic myeloid leukemia (CML) in male patients and female patients (2007–2023). The line graph shows yearly incidence rates of CML stratified by sex: female patients (blue), male patients (orange), and total combined (gray). Data are represented as incidence per 100,000 population. Both absolute numbers (N) and percentages (%) were used to calculate incidence. Statistical comparisons between sexes were performed using the chi-square test, with p-values <0.05 considered statistically significant. The graph highlights a consistent predominance of female cases compared with male patients throughout the study period, particularly after 2010. Data are expressed as incidence rates per 100,000 population. Both absolute case numbers (N) and percentages (%) were used for calculations.

**Figure 3:**
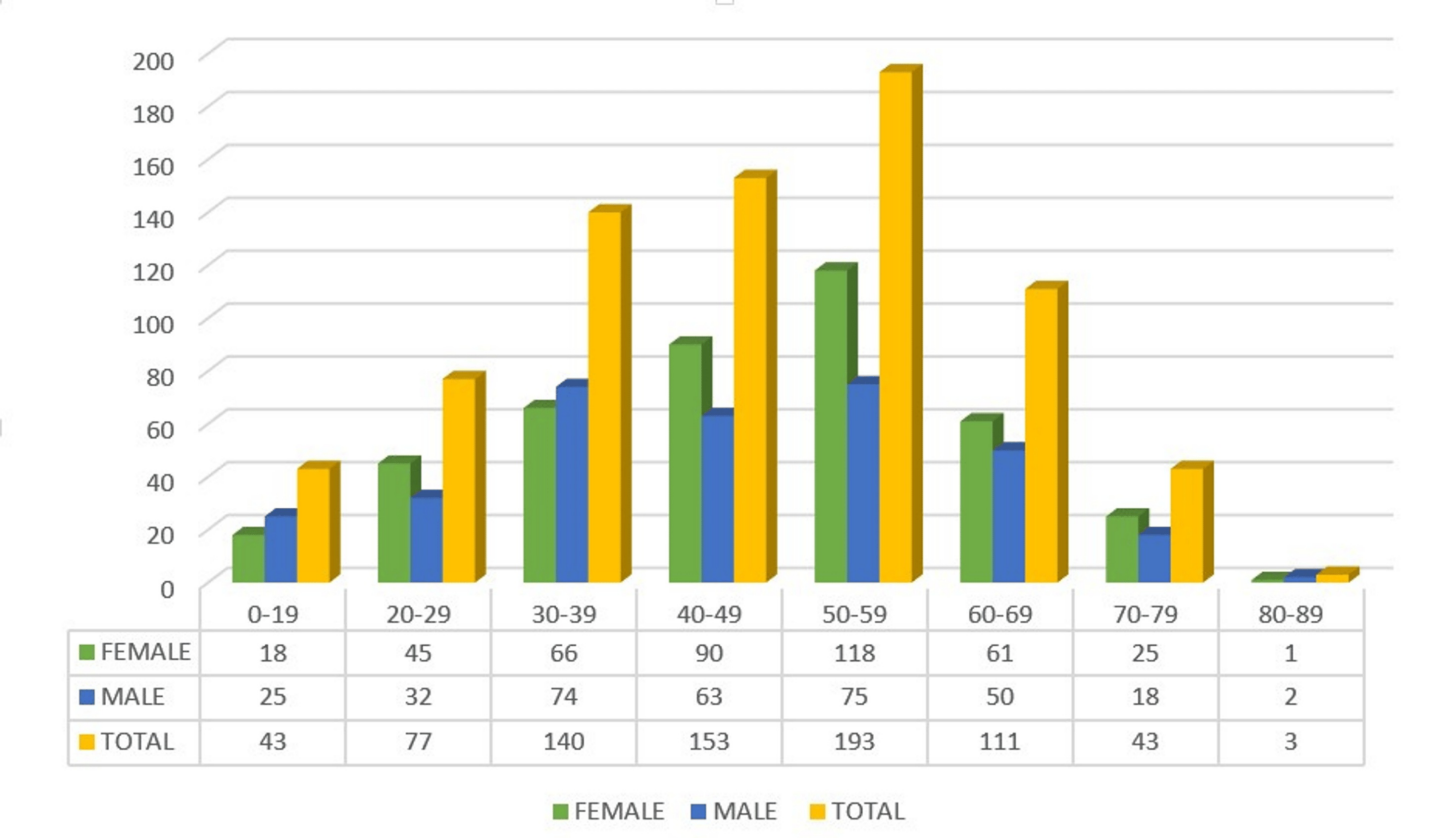
Age and gender distribution of chronic myeloid leukemia (CML) patients. The bar chart shows absolute case numbers (N) for each age group; percentages (%) relative to the total population are provided below the figure. Green bars represent female patients, blue bars male patients, and yellow bars the combined total. Corresponding values (N) are provided below the figure. The majority of patients were diagnosed between 40 and 59 years, with peak incidence observed in the 50–59 age group (female patients: n=118; male patients: n=75; total: n=193). Data are presented as absolute numbers (N) and percentages (%) of the study population. No statistical tests were applied; values are descriptive only.

**Figure 4:**
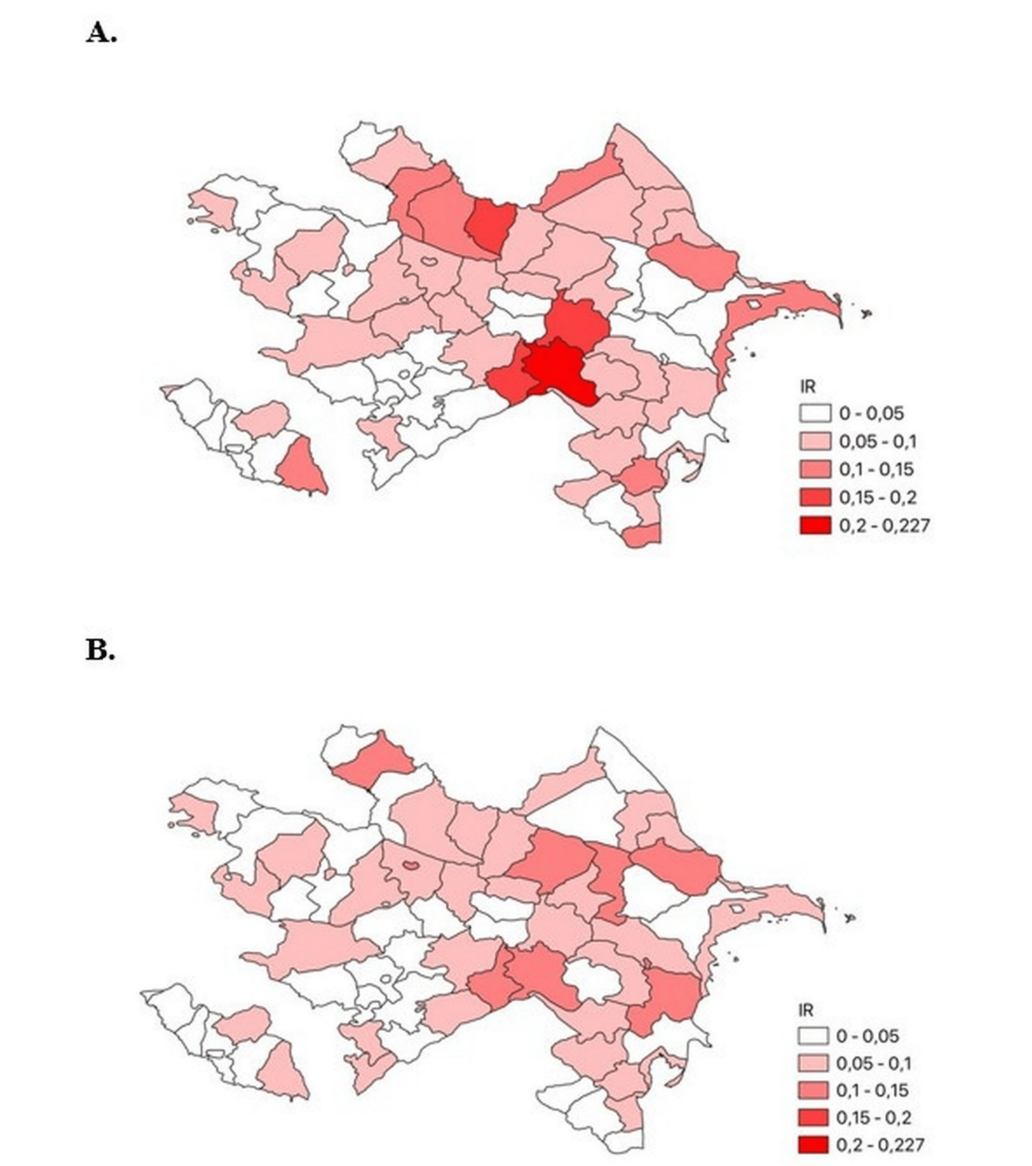
Regional distribution of chronic myeloid leukemia (CML) patients in Azerbaijan by sex. (A) Female patients and (B) Male patients. The choropleth maps display the regional distribution of CML incidence across Azerbaijan, expressed as cases per 100,000 population. To facilitate comparison, female and male incidence rates are presented as separate maps using identical scales. This design minimizes the risk of confusion that could arise from combining two color schemes in a single map. Darker shades represent higher incidence (0.20–0.27 per 100,000), while lighter shades correspond to lower incidence (<0.05 per 100,000). The highest concentration of female patients is observed in Baku and several northern districts, whereas the distribution of male patients is more evenly spread across regions. Data are expressed as incidence rates per 100,000 population. Shading intensity corresponds to case frequency relative to the regional population (%). No statistical tests were applied.

Our data confirm a female predominance of CML in Azerbaijan, contrasting with the global male predominance. This finding, consistent across most age groups and regions, suggests possible influences of local environmental exposures (e.g., petrochemical industries) and diagnostic practices. Of the 763 patients diagnosed with CML in Azerbaijan between 2008 and 2023, 54% were female and 46% were male-contrasting with the global pattern of male predominance. The incidence rate was 0.4 per 100,000 for women and 0.34 for men, with female predominance observed across nearly all age groups except zero-nineteen, thirty-thirty-nine, and eighty-eighty-nine years. The value of 0.34 shown in Figure 1 corresponds to the incidence rate in male patients. The number of newly diagnosed CML cases increased 3.2-fold over the study period, rising from 27 cases in 2008 to a peak of ninety-nine in 2016. The median age at diagnosis was 46.6 years for female patients and 44.4 years for male patients. Regionally, female cases were most common in the Ganja-Kazakh zone (a western administrative region of Azerbaijan), while male predominance was seen in Aran (a central lowland region). The highest absolute number of cases was recorded in the Absheron region (which includes the capital Baku and surrounding industrial areas), an area of intense industrial and petrochemical activity (Figures 1-4). For clearer visualization, regional incidence was displayed as two separate choropleth maps for female patients and male patients (Figure 4). This side-by-side approach maintains a consistent color scale for each sex, ensuring accurate interpretation of regional variation and female predominance.

Clinical characteristics and imatinib response

There were no significant sex-based differences in CML phase or risk category at diagnosis. However, female patients presented with significantly smaller spleen sizes (mean 6.16 ± 4.6 cm vs. 7.35 ± 5.0 cm in male patients, p = 0.00001), lower hemoglobin levels (92.9 ± 20.1 g/L vs. 97.9 ± 24.9 g/L), and markedly higher platelet counts (384 × 10⁹/L vs. 246 × 10⁹/L, p = 0.00001). Rh-negative blood type was significantly more frequent among female patients (p = 0.011). No statistically significant differences were observed in white blood cell count or peripheral blast percentages between the sexes.

A total of 85.2% of all patients achieved major molecular response (MMR) following imatinib treatment. Among those who achieved MMR, 55.2% were female and 44.8% were male, indicating a significantly better treatment response in female patients (p = 0.03) (Table 1).

**Table 1:** Gender-based clinical characteristics and imatinib response in chronic myeloid leukemia (CML) patients. The table summarizes baseline clinical features and treatment response in 763 patients with CML, stratified by sex (female, n=409; male, n=354). Data are presented as absolute numbers (N) with row percentages (%) in brackets. Statistical comparisons between sexes were performed using chi-square (χ²) tests, with p<0.05 considered significant. CML phase: Most patients were diagnosed in the chronic phase (574/763; 75.2%), with no significant sex differences (χ²=1.475, p=0.478). Sokal risk group: The majority were in the intermediate-risk category (415/763; 54.4%), without sex-related differences (χ²=1.261, p=0.532). Spleen size: Male patients had a significantly higher frequency of massive splenomegaly (>8 cm) compared with female patients (128/354 vs. 85/409; χ²=22.35, p=0.00001). Hemoglobin (Hb): Anemia was common, with moderate anemia (80–99 g/L) being the most frequent (290/763; 38.0%). Male patients had significantly more normal Hb values than female patients, while female patients had higher rates of moderate/severe anemia (χ²=15.09, p=0.0017). White blood cell (WBC) count: Most patients presented with leukocytosis (>9.0 ×10⁹/L, 728/763; 95.4%), with no sex difference (χ²=0.932, p=0.627). Platelets (PLT): Platelet abnormalities differed significantly by sex. Thrombocytopenia was more common in males (80/354, 22.6%), while thrombocytosis was slightly more frequent in females (98/409, 23.9%) (χ²=122.329, p=0.00001). Peripheral blasts: The majority had 1–10% peripheral blasts (403/763; 52.8%) with no sex-related difference (χ²=3.931, p=0.140). Imatinib response: Females had higher response rates (359/409, 87.8%) compared with males (291/354, 82.2%), reaching statistical significance (χ²=4.669, p=0.030). Blood group: Distribution was similar between sexes, with blood group A most common (328/763; 43.0%) (χ²=6.2, p=0.102). Rh factor: A significantly higher proportion of females were Rh-negative (44/409, 10.8%) compared with males (20/354, 5.6%) (χ²=6.4, p=0.011).

Category	Total (N=763)	Female (N=409)	Male (N=354)	p-value (χ²)	
CML phase		-	-	Chi=1.475 P=0.478	
Chronic	574 (100%)	307 (53.5%)	267 (46.5%)	-	-
Accelerated	130 (100%)	74 (56.9%)	56 (43.1%)	-	-
Blast Crisis	59 (100%)	28 (47.5%)	31 (52.5%)	-	-
Sokal risk group		-	-	Chi=1.261 P=0.532	
High	136 (100%)	71 (52.2%)	65 (47.8%)	-	-
Intermediate	415 (100%)	230 (55.4%)	185 (44.6%)	-	-
Low	212 (100%)	108 (50.9%)	104 (49.1%)	-	-
Spleen	-	-	-	Chi=22.35 P=0.00001	
Normal	42 (100%)	24 (57.1%)	18 (42.9%)	-	-
Moderate (≤8 cm)	508 (100%)	300 (59.1%)	208 (40.9%)	-	-
Massive (>8 cm)	213 (100%)	85 (39.9%)	128 (60.1%)	-	-
Hgb (g/l)	-	-	-	Chi=15.09 P=0.0017	
Normal (120-160 g/l)	109 (100%)	40 (36.7%)	69 (63.3%)	-	-
Mild anemia (100-119 g/l)	194 (100%)	106 (54.6%)	88 (45.4%)	-	-
Moderate anemia (80-99 g/l)	290 (100%)	164 (56.6%)	126 (43.4%)	-	-
Severe anemia (65-79 g/l)	170 (100%)	99 (58.2%)	71 (41.8%)	-	-
WBC (х10⁹/l)		-	-	Chi=0.932 P=0.627	
Normal (3.9-9.0)	32 (100%)	19 (59.4%)	13 (40.6%)	-	-
Leukopenia (<3.9)	3 (100%)	1 (33.3%)	2 (66.7%)	-	-
Leukocytosis (>9.0)	728 (100%)	389 (53.4%)	339 (46.6%)	-	-
PLT (х10⁹/l)		-	-	Chi=122.329 P=0.00001	
Normal (180-400)	439 (100%)	241 (54.9%)	198 (45.1%)	-	-
Thrombocytopenia (<180)	150 (100%)	70 (46.7%)	80 (53.3%)	-	-
Thrombocytosis (>400)	174 (100%)	98 (56.3%)	76 (43.7%)	-	-
Peripheral blast cells			-	Chi=3.931 P=0.140	
0	340 (100%)	173 (50.9%)	167 (49.1%)	-	-
1-10	403 (100%)	228 (56.6%)	175 (43.4%)	-	-
>10	20 (100%)	8 (40.0%)	12 (60.0%)	-	-
Imatinib response		-	-	Chi=4.669 P=0.030	
Responders	650 (100%)	359 (55.2%)	291 (44.8%)	-	-
Resistants	113 (100%)	50 (44.2%)	63 (55.8%)	-	-
Blood type		-	-	Chi=6.2 P=0.102	
0	265 (100%)	141 (53.2%)	124 (46.8%)	-	-
A	328 (100%)	164 (50.0%)	164 (50.0%)	-	-
B	109 (100%)	69 (63.3%)	40 (36.7%)	-	-
AB	61 (100%)	35 (57.4%)	26 42.6%)	-	-
Rhesus factor		-	-	Chi=6.4 P=0.011	
Positive	699 (100%)	365 (52.2%)	334 (47.8%)	-	-
Negative	64 (100%)	44 (68.8%)	20 (31.2%)	-	-

Molecular characteristics

A key finding is the significantly higher susceptibility of male patients to ABL kinase domain mutations, especially T315I, which underscores the importance of sex-informed genetic profiling and closer molecular monitoring in this group. Of the 163 patients with available molecular data, ABL kinase domain mutations were identified in 22 cases (13.5%). These mutations were significantly more common among male patients (21.3%) compared to female patients (6.0%) (p = 0.004, χ² = 8.08). The most frequent mutation was T315I, found in seven patients overall: five male patients (35.7% of male mutations) and two female patients (40% of female mutations). Other recurrent mutations included F359C (three cases: two male patients, one female patient), F359V (three cases: two male patients, one female patient), F317L in two forms-TTG (two cases: one male patient, one female patient) and TTA (one male patient), Y253H (two male patients), and E255K (one male patient). Female patients carried only a limited subset of these mutations, with F359C, F317L (TTG), F359V, and T315I being the only variants identified among them. This pattern indicates a broader mutation spectrum and higher mutation burden among male patients.

Additional chromosomal aberrations (ACAs) were detected in thirty-one patients (19% of those tested), with a higher frequency in male patients (24.3%) compared to female patients (14.6%), although this difference was not statistically significant (p = 0.115, χ² = 2.47). Importantly, eight male patients had both ACA and ABL mutations concurrently, compared to only one female, suggesting a potentially more complex cytogenetic profile and cumulative genetic risk in male patients.

Analysis of cytochrome P450 gene polymorphisms (CYP3A53, CYP3A418, CYP2B6*6) revealed no significant sex-based differences, consistent with prior pharmacogenomic studies. However, functional differences in CYP enzyme activity and drug transporter expression may still influence treatment pharmacokinetics and toxicity profiles, particularly in male patients (Tables 2, 3).

**Table 2:** ABL kinase domain mutations and additional chromosome aberrations (ACAs) in chronic myeloid leukemia (CML) patients by sex. The table summarizes the frequency of ABL kinase domain mutations and additional chromosome aberrations in male and female patients. Data are presented as absolute numbers (N) and percentages (%). Statistical comparisons between sexes were performed using chi-square (χ²) tests, with p<0.05 considered significant. ABL kinase domain mutations were observed more frequently in male patients (17/80; 21.3%) than female patients (5/83; 6.0%) (χ²=8.08, p<0.004). ACAs were present in both sexes with similar frequency: female patients (13/89; 14.6%) and male patients (18/74; 24.3%), including cases with coexisting ABL mutations (female: 1; male: 8), with no significant sex difference (χ²=2.47, p=0.115).

Gender	Total (163)	With	Without	p-value
ABL kinase domain mutations			-	-
Female	83	5	78	<0.004
Male	80	17	63	Chi=8.08
Additional chromosome aberration				-
Female	89	13 (1 with mutation)	76	<0.115
Male	74	18 (8 with mutation)	56	Chi=2.47

**Table 3:** Distribution of CYP3A53, CYP3A418, and CYP2B6*6 genotypes and allele frequencies in patients and controls, stratified by sex and age. Data are presented as absolute numbers (N) and percentages (%), with allele frequencies calculated accordingly. For CYP3A53, the GG genotype predominated in both patients (99.3%) and controls (95%), while the A allele was more frequent in patients (p=0.015). CYP3A418 showed a predominance of the TT genotype in both patients (98%) and controls (100%), with no significant differences in allele distribution (p=0.159). For CYP2B6*6, the GG, GT, and TT genotypes were distributed similarly in patients and controls, with no significant differences by sex or age (χ²=2.472, p=0.291). Statistical comparisons were performed using chi-square (χ²) tests, with p<0.05 considered statistically significant.

CYP3A5*3 Frequency Parameter	Genotypes, n (%) AA	Genotypes, n (%) AG	Genotypes, n (%) GG	Alleles A	Alleles G	p-value
Patients	1 (0.7)	–	152 (99.3)	0.007	0.993	p=0.015
Control group	–	5 (5)	95 (95)	0.025	0.975	–
Male	1 (1.3)	–	78 (98.7)	0.02	0.98	–
Female	–	–	74 (100)	0	1	p=0.869
<20	–	–	7 (100)	0	1	p=0.109
20–40	1 (2)	–	53 (98)	0.02	0.98	–
>40	–	–	92 (100)	0	1	–
CYP3A4*18 Frequency Parameter	Genotypes, n (%) TT	Genotypes, n (%) TC	Genotypes, n (%) CC	Alleles T	Alleles C	p-value (χ²)
Patients	150 (98)	3 (2)	–	0.99	0.01	p=0.159
Control group	100 (100)	–	–	1	0	–
Male	77 (98)	2 (2)	–	0.98	0.02	p=0.605
Female	73 (99)	1 (1)	–	0.99	0.01	–
<20	7 (100)	–	–	1	0	–
20–40	52 (97)	2 (3)	–	0.97	0.03	p=0.109
>40	91 (99)	1 (1)	–	0.99	0.01	–
CYP2B6*6 Frequency Parameter	Genotypes, n (%) GG	Genotypes, n (%) GT	Genotypes, n (%) TT	Alleles G	Alleles T	p-value (χ²)
Control group	74 (48.4)	54 (35.3)	25 (16.3)	0.65	0.35	p=0.291, χ²=2.472
Male (n=79)	43 (54.5)	23 (29)	13 (16.5)	0.69	0.31	p=0.257, χ²=2.71
Female (n=74)	31 (41)	30 (40)	13 (19)	0.61	0.39	–
<20	4 (57)	2 (28)	1 (15)	0.71	0.29	p=0.974
20–40	29 (53)	15 (28)	10 (19)	0.68	0.32	–
>40	45 (48)	28 (30)	19 (22)	0.63	0.37	–

Survival and mortality

**Figure 5:**
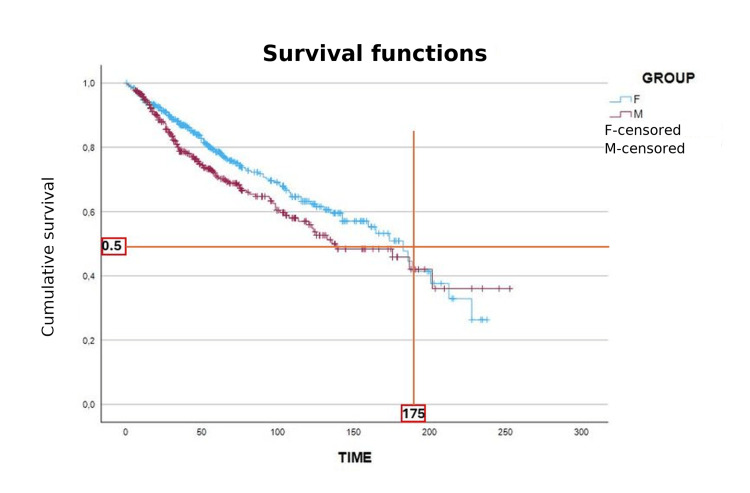
Overall survival (OS) of chronic myeloid leukemia (CML) patients by sex (Kaplan–Meier analysis). The Kaplan–Meier survival curves compare overall survival between female (blue line) and male (red line) CML patients. Median OS was longer in female patients (≈175 months) compared to male patients. Survival probability at 50% (0.5) is indicated by the horizontal reference line, while censored cases are shown as tick marks. Data are expressed as survival probability over time (months). Comparison between groups was performed using the log-rank test, which demonstrated significantly better survival in female patients (p=0.038).

**Figure 6:**
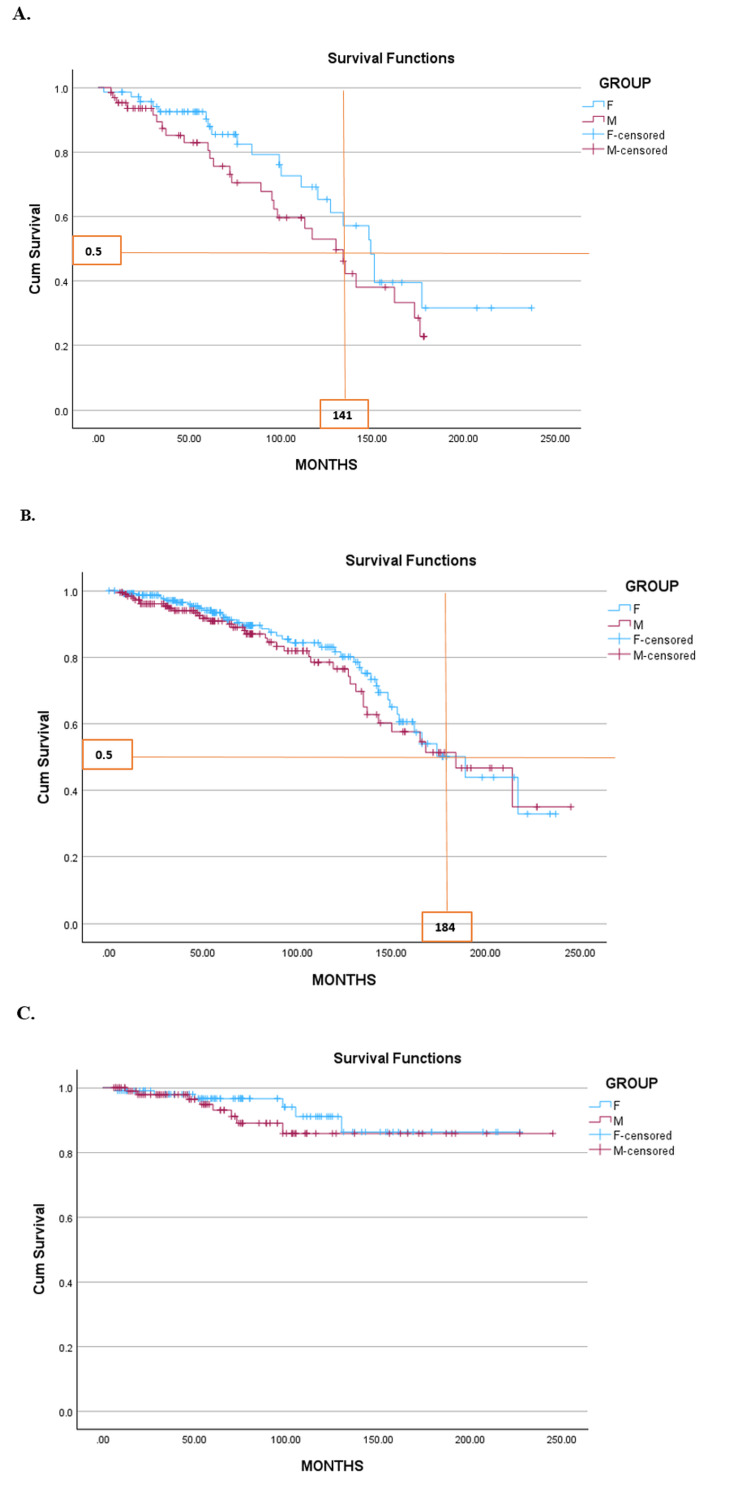
The survival rate by sex of patients with chronic myeloid leukemia (CML) according to risk groups (Kaplan-Meier). (A) High-risk group, (B) Intermediate-risk group, and (C) Low-risk group Kaplan–Meier survival curves are shown for female patients (blue) and male patients (red) stratified by prognostic risk: (A) High-risk group, (B) Intermediate-risk group, and (C) Low-risk group. Data are presented as survival probability over time (months), with censored cases indicated by tick marks. In the high-risk subgroup, median OS was 149 months for female patients and 130 months for male patients; this difference did not reach statistical significance (log-rank p=0.185), likely due to limited sample size. In the intermediate-risk subgroup, survival was nearly identical (female patients: 189 months, male patients: 184 months; p=0.460). In the low-risk subgroup, both sexes demonstrated excellent long-term survival with no significant difference (p=0.379). For all comparisons, p<0.05 was considered statistically significant.

Importantly, while female patients demonstrated superior overall survival, our analysis also shows that TKI therapy largely neutralizes sex-based survival disparities in low- and intermediate-risk groups, supporting the robustness of current treatment strategies. Kaplan-Meier survival analysis showed significantly better overall survival for female patients, with a median of 175 months, compared to male patients (log-rank p = 0.038) (Figure 5). In the high-risk subgroup, median survival was 149 months for female patients and 130 months for male patients, though this difference did not reach statistical significance (p = 0.185), likely due to sample size limitations. In intermediate-risk patients, survival was nearly identical between sexes (189 vs. 184 months; p = 0.460), and in the low-risk group, both sexes demonstrated excellent survival with no significant difference (p = 0.379) (Figure 6).

The mortality-to-incidence ratio (MIR) declined substantially from 0.0025 in 2009 to below 0.001 by 2023, reflecting improved access to diagnostic and treatment services. Until 2017, the MIR was consistently higher in male patients but subsequently declined to converge with that of female patients. By 2022, the MIR was virtually equal between sexes, suggesting improved equity in clinical outcomes over time.

## Discussion

Our study revealed a unique sex distribution of CML in Azerbaijan, with female patients (54%) outnumbering male patients (46%), in contrast to the male predominance reported in most global analyses. The unexpected female predominance observed in our cohort may be influenced by regional environmental exposures, including high petrochemical activity in industrial zones, combined with population-level genetic predispositions. Furthermore, national cancer registry data from Azerbaijan and neighboring countries have also documented higher female rates in certain hematologic malignancies, suggesting that diagnostic patterns and environmental cofactors could contribute. One possible explanation may be a higher number of target cells in women, alongside hormonal or environmental factors [13,14]. Estrogen receptor signaling has been shown to influence apoptotic pathways and gene expression in leukemic cells, which may partially explain better responses and outcomes in women [15]. In vitro studies suggest that estrogen activation reduces the viability of BCR-ABL1-positive hematopoietic progenitors and suppresses leukemic stem cell clonogenicity [13,16], offering a rationale for exploring combined hormonal-TKI therapy in resistant CML. Although these data remain preclinical, they support the hypothesis that estrogen may have a protective role in CML.

Our findings are further supported by national data from the State Statistical Committee of Azerbaijan, which shows female predominance in malignancies overall. Similar CML incidence patterns were also reported in Turkey and France. A large EUTOS registry of 2,904 CML patients across 20 European countries found comparable trends [17], as did a Turkish retrospective analysis [18].

Over the study period (2008-2023), CML incidence in Azerbaijan increased 3.2-fold, reflecting population growth, improved diagnostic infrastructure, and enhanced care, in line with global trends. Despite variability in annual counts, this trend mirrors the global 54.1% increase in CML cases between 1990 and 2019, although the age-standardized incidence rate declined slightly [19].

Our age- and sex-stratified analysis showed similar median ages across sexes, but a higher female incidence across most age groups. Regionally, female cases predominated in Ganja-Kazakh, while males were more frequent in Aran. The highest overall CML frequency was observed in Absheron, a heavily industrialized zone with petrochemical exposure [14], suggesting possible environmental interactions with sex-specific risk factors.

Clinically, female patients had smaller spleens, lower hemoglobin, and higher platelet counts, findings consistent with previous reports [7,20]. Female patients also demonstrated higher imatinib response rates and superior survival, which may be partly explained by estrogen-mediated effects on leukemic stem cells and immune modulation. Experimental studies have shown that estrogen signaling can induce apoptosis in BCR-ABL-positive progenitors and reduce leukemic stem cell clonogenicity, offering a mechanistic rationale for this advantage. These differences likely reflect physiological patterns (e.g., smaller spleen size, higher platelet levels, and iron deficiency in female patients). We also found an association between Rh-negative status and female sex (p = 0.011), which may reflect population-specific genetic factors.

Platelet biology is influenced by sex, with females typically having higher counts and enhanced activation [21]. Treatment response analysis showed that more females achieved MMR at 12 months, aligning with global studies showing sex-related differences in imatinib response [22-24].

Molecular analyses further highlighted important disparities. The higher frequency of ABL kinase domain mutations in male patients may reflect sex-related biological susceptibility, possibly linked to differential drug metabolism, pharmacogenomic factors, or immune surveillance. Prior studies have also suggested that adherence to therapy and pharmacokinetic variability may differ by sex, which could contribute to the observed pattern. Among 163 patients assessed for mutations, male patients had significantly higher rates of ABL kinase mutations (21.3% vs. 6%, p = 0.004), including the T315I mutation (9.8% vs. 3.1%, p = 0.036) [25]. These findings support the need for sex-informed genetic profiling and molecular monitoring, especially in male patients. While mutation frequency differed by sex, ACAs did not show significant sex association (p = 0.115), despite being more common in male patients (24.3% vs. 14.6%), consistent with prior literature [26].

Cytochrome P450 polymorphisms (CYP3A53, CYP3A418, CYP2B6*6) showed no significant sex-related differences, although enzyme activity and drug transporter expression may still influence treatment efficacy [27,28].

The higher mutation rate in male patients may reflect sex-specific biological susceptibility. Prior studies suggest mutation risk can be influenced by immune status, drug metabolism, or adherence [29,30]. Naidoo et al. proposed that sex-related CYP polymorphisms might impact intracellular drug levels and resistance [31].

While more male patients had both ACAs and ABL mutations, the lack of statistical significance limits firm conclusions. Nonetheless, the co-occurrence of these abnormalities warrants further exploration in larger, multicenter studies.

Survival analysis demonstrated significantly better overall survival in female patients (p = 0.038), consistent with previous findings suggesting sex influences treatment response and progression [26,27]. Importantly, our analysis shows that modern TKI therapy largely neutralizes sex-related disparities in survival for low- and intermediate-risk groups. This suggests that, while biological differences exist, the efficacy of imatinib and related TKIs is sufficient to overcome most baseline sex-associated disadvantages. Estrogen’s immunomodulatory and apoptotic effects may contribute [23]. In high-risk patients, female patients lived 19 months longer (149 vs. 130 months), though this was not statistically significant (p = 0.185), likely due to the limited sample size. Notably, survival scores such as Sokal and EUTOS do not incorporate sex, despite emerging evidence of its prognostic value [32].

In intermediate-risk patients, survival was similar (189 vs. 184 months; p = 0.460), indicating that TKIs may mitigate sex differences in this group. Prior studies suggest that other variables, such as age, spleen size, and platelet counts, are stronger predictors than sex [25]. Kaplan-Meier curves were nearly identical, supporting this observation.

In low-risk CML, both sexes achieved excellent long-term survival (p = 0.379), with curves plateauing above 85 percent, in agreement with previous findings [32,33]. These results suggest that modern TKI therapy neutralizes most sex-related differences in low- and intermediate-risk CML.

The marked reduction in the MIR in both sexes over the past two decades reflects the success of TKI therapy [32,33]. The slight female survival advantage may stem from behavioral or pharmacogenetic factors [34]. By 2020-2023, survival outcomes between sexes converged, likely due to improved and more equitable care access. Sustained surveillance and sex-specific research remain essential to further optimize outcomes.

Strengths and limitations

This study has several notable strengths. It represents the largest retrospective cohort of CML patients analyzed in Azerbaijan to date, providing valuable real-world insight from a population that is underrepresented in global CML registries. The use of a uniform diagnostic and therapeutic framework from a single national center reduces heterogeneity in patient management. Furthermore, the focused gender-stratified analyses provide novel epidemiological and molecular insights with potential clinical relevance.

Nonetheless, certain limitations must be acknowledged. As with all retrospective studies, the potential for selection bias and incomplete data cannot be excluded. Although we defined “complete data” rigorously and excluded patients with missing key variables, this may still limit inclusiveness. Some potential confounders, such as adherence to therapy, hormonal status, or environmental exposures, were not systematically captured. The single-country, single-center design may also restrict generalizability beyond the Azerbaijani population. Finally, although the sample size is substantial for a national cohort, subgroup analyses (e.g., mutations by sex) were limited by smaller numbers.

Another limitation is the lack of systematic assessment of patient adherence to TKI therapy and the absence of imatinib plasma trough level monitoring. Both factors may influence resistance patterns and survival outcomes, and their omission restricts a more nuanced interpretation of treatment response. Furthermore, although molecular testing and statistical analyses were performed according to established international laboratory and analytical standards, detailed step-by-step protocols (e.g., assay conditions, sequencing specifications) were not reported, which may limit replicability in other settings.

Despite these limitations, the consistency of observed trends and the large patient population strengthen the reliability of our findings.

## Conclusions

This nationwide study reveals striking sex-related disparities among patients with CML in Azerbaijan. Female patients not only had a higher incidence but also presented with more favorable clinical features and superior overall survival. In contrast, male patients exhibited a significantly higher frequency of resistance-associated ABL kinase domain mutations, which may underlie their comparatively poorer outcomes. No significant sex-based differences were observed in CYP gene polymorphisms.

These findings underscore the critical importance of incorporating sex-specific considerations into CML diagnosis, prognostic scoring, and therapeutic decision-making. Enhanced molecular monitoring, particularly in male patients, and personalized treatment approaches may improve outcomes. Acknowledging sex-related biological differences can guide more tailored research and improve equity in CML management.
